# Current practice and novel approaches in organ preservation

**DOI:** 10.3389/frtra.2023.1156845

**Published:** 2023-06-08

**Authors:** Ozge Sila Ozgur, Bat-Erdene Namsrai, Timothy L. Pruett, John C. Bischof, Mehmet Toner, Erik B. Finger, Korkut Uygun

**Affiliations:** ^1^ Department of Surgery, Center for Engineering in Medicine and Surgery, Massachusetts General Hospital, Harvard Medical School, Boston, MA, United States; ^2^ Research Department, Shriners Children’s Boston, Boston, MA, United States; ^3^ Department of Surgery, University of Minnesota, Minneapolis, MN, United States; ^4^ Departments of Mechanical and Biomedical Engineering, University of Minnesota, Minneapolis, MN, United States

**Keywords:** organ transplantation, liver preservation, machine perfusion, subzero preservation, nanowarming, supercooling, isochoric preservation, partial freezing

## Abstract

Organ transplantation remains the only treatment option for patients with end-stage organ failure. The last decade has seen a flurry of activity in improving organ preservation technologies, which promise to increase utilization in a dramatic fashion. They also bring the promise of extending the preservation duration significantly, which opens the doors to sharing organs across local and international boundaries and transforms the field. In this work, we review the recent literature on machine perfusion of livers across various protocols in development and clinical use, in the context of extending the preservation duration. We then review the next generation of technologies that have the potential to further extend the limits and open the door to banking organs, including supercooling, partial freezing, and nanowarming, and outline the opportunities arising in the field for researchers in the short and long term.

## 1. Introduction

Organ transplantation remains the only treatment option for patients with end-stage organ failure. If combined, the number of deaths due to organ failure exceeds cancer and all other causes (1). The discrepancy between patient need and the number of organs available results in a transplant waiting list of over 100,000 in the United States alone, which is generally considered the tip of the iceberg for those in need. Geographic differences in organ availability and other factors further lead to inequity in access to transplant medicine (2). Although there has been rapid growth in organ transplantation since 2013, and a record number of total solid organ transplants were performed in 2020, the need for more transplantable grafts remains desperately high (2, 3).

**Figure 1:**
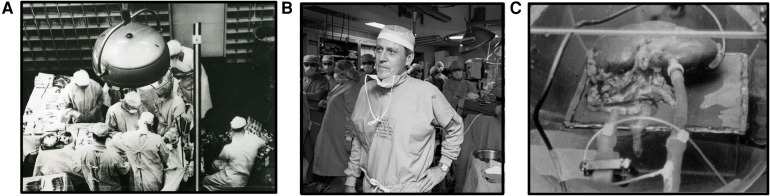
The evolution of machine perfusion. (**A**) Dr. Joseph Murray, while performing the first successful organ transplantation, 1954 (113). (**B**) Dr. Thomas Starzl, after performing a transplant surgery (115). (**C**) Dr. Folkert Belzer's kidney perfusion machine (116).

Clinical transplantation began with the pioneering work of Joseph Murray, who performed the first successful organ transplantation in 1954 (Figure 1A) (4). In 1967, Thomas Starzl performed the first successful liver transplantation under immunosuppression, with survival exceeding 1 year (Figure 1B) (5). Once a solution for immune rejection was identified, preserving organs in a viable, transplantable condition for an extended period became the key technological barrier for providing access to this life-saving treatment (1). As noted by Southard and Belzer, who developed the University of Wisconsin organ preservation solution, preservation technology is “the supply line for organ transplantation” (6). In its current state, organ transplants can be described as a unique supply chain management problem, where the stakes are simply the lives of the patients.

In the 1960s, Belzer developed the first hypothermic machine perfusion (HMP) device that was clinically applicable (Figure 1C) and performed the first HMP-preserved human kidney transplantation in 1967 (7). However, this method was considered expensive and difficult to implement at the time. In 1969, Collins et al. started the era of cold storage by demonstrating the successful transplantation of canine kidneys after storage in a small box with ice for 30 h (8). The introduction of the University of Wisconsin solution in the 1980s by Belzer and Southard represented a breakthrough in organ preservation (9). Static cold storage (SCS) replaced machine perfusion and became the clinical gold standard, enabling organ transplantation to be a vast success that can reach very high success rates—unlike those early days when it was seen as a last resort.

**Figure 2:**
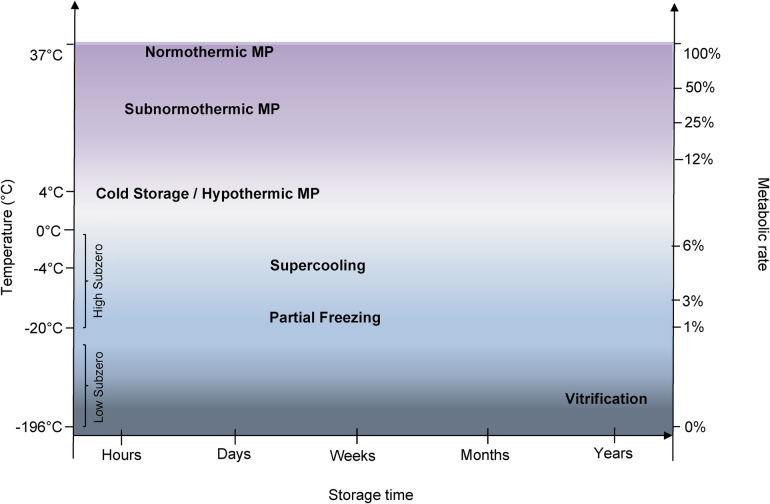
Key organ preservation techniques and the interplay of temperature and metabolic rates. Metabolic rates are estimated based on the van't Hoff rule, which estimates the metabolic rate approximately doubles with every 10°C increase in temperature (115).

The last decade has seen a flurry of activity in improving organ-preservation technologies (Figure 2): machine perfusion, abandoned in the 1980s due to cost and practicality concerns, has made a dramatic comeback and is now permeating into the operating rooms (ORs) rapidly (10, 11). In parallel, there is a convergent arch of major technological development in the field of cryobiology, which was long limited to cells and reproductive medicine, now offering many innovative alternatives for long-term organ preservation in subzero temperatures. Perhaps, surprisingly, these new cryobiology approaches critically leverage machine perfusion as a platform technology and build on its success to enable these next-generation methods. In this review, we present a brief update on the developments, achievements, and innovations that have taken place in the field of extended organ preservation over approximately the last 10 years. To make the task tractable, we focus primarily on liver preservation. However, we have allowed limited side notes to other organ systems where horizontal translation of technologies appears obvious, where appropriate.

## 2. Static cold storage

Cold storage has been an enabling technology that has allowed transplantation to be an accessible treatment for organ failure. Even though this method is very practical and cost-effective, it is insufficient for the following three key reasons: (1) the organs actively suffocate during storage, and even the high-quality organs can only be stored for a few hours (12); (2) non-ideal donors organs, particularly extended criteria donors (ECDs), have a higher sensitivity to cold ischemia (13) exacerbating the ischemia-reperfusion injury (IRI) (14). Briefly, when the organs are deprived of oxygen, mitochondria function and adenosine triphosphate levels decrease, resulting in cellular swelling and apoptosis. After organs are reperfused, cell injury is aggravated by triggering the formation of reactive oxygen species (ROS) and immune response, which is associated with delayed graft function and primary graft failure (15). In practice, this means that marginal organs often present a higher risk of failure, and many of these are not used; (3) since the organ is not fully functional at ice-cold temperatures, it is difficult to assess if it will function as expected. To avoid the risk of primary non-function, population statistics-based approaches, such as the Donor Risk Index, are used to select higher-quality donor organs. But, by their nature, these scoring systems are conservative, and many potentially transplantable organs are not used (16). These disadvantages led to the development of alternative preservation techniques, leading to the rise of an old, almost forgotten process that Murray himself had actually used.

## 3. Oxygenated machine perfusion

As the number of ideal organs is insufficient to meet the transplant demand, marginal organs, particularly those from donors after cardiac death (DCD), have become one approach to expand the donor organ pool. For DCD organs, the method for preservation is more important than heart-beating donor organs [donors after brain death (DBD)] because there is already an existing level of warm ischemic injury and a higher risk for graft malfunction (17). Machine perfusion continuously provides oxygen and nutrients, which enables the graft to restore tissue energy (18), thus allowing a certain amount of repair and recovery of an ischemic organ ex situ before transplantation (19). As a functional preservation modality, it also allows full organ functional assessment, in contrast to SCS, which effectively only slows the rate of ischemic injury. The operation temperature of machine perfusion can be simplistically divided into three main categories: HMP at 4°C–10°C; subnormothermic machine perfusion (SNMP; also referred to as mid-thermic perfusion) at 20°C–33°C; and normothermic machine perfusion (NMP) at 36°C–38°C, depending on the species used in the study (a more rigorous breakdown including recommendations for standardized terminology can be found in Karangwa et al. (20)). Since perfusion is synergistic with all extended preservation approaches, and itself allows significant opportunities to extend preservation beyond the current clinical limits, we first summarize key recent studies in liver machine perfusion (Table 1).

**Table 1:** A brief summary of key studies in liver perfusion categorized based on operating temperatures.

Author	Year	Replicates in key groups	Type	WIT	CIT	Oxygenated perfusion time	Perfusate	Temperature	Endpoints	Outcomes
*HMP*										
Guarrera et al. (22)	2010	20	Human liver	44.3 ± 6.5 min	9.4 ± 2.1 h	4.3 ± 0.9 h	Vasosol with added antioxidants, metabolic substrates, and vasodilators	HMP (4°C–6°C)	Mean incidences of primary non-function, early allograft dysfunction, and patient and graft survival at 1 month and 1 year	No cases of primary non-function. Early allograft dysfunction rates were 5% in the HMP group vs. 25% in controls, no vascular complications, serum injury markers were significantly lower
Bruggenwirth et al. (30)	2020	Group 1: 6	Porcine liver	30 min	2 h	Group 1: 2 h	Belzer UW machine perfusion solution oxygenated with 100% O_2_	HMP (DHOPE)	Lactate clearance, glucose, ALT, LDH, HMGB-1, IL-6, TNFα, cfDNA, bile duct, and liver histology	Biliary pH, bicarbonate, and LDH were similar in all groups at the end of reperfusion. Levels of ALT, HMGB-1, or cfDNA in the perfusate were not elevated by extended HMP (DHOPE). Histological analysis of liver parenchyma revealed that HMP (DHOPE) for up to 24 h did not result in more necrosis after reperfusion compared to shorter preservation times
		Group 2: 6				Group 2: 6 h				
		Group 3: 6				Group 3: 24 h				
Van Rijn et al. (29)	2021	78	Human liver	–	6 h 11 min (median)	2 h 12 min (median)	Cold Belzer machine perfusion solution, supplemented with glutathione	HMP (DHOPE) (10°C)	Serum markers of hepatobiliary injury and function, intraoperative postreperfusion syndrome	Lower risk of non-anastomotic biliary strictures (6%) and postreperfusion syndrome (12%) compared to the CS group
*SNMP*										
Berendsen et al. (38)	2012	Group 1: 6	Rat liver	Group 1: 1 h	–	3 h	Williams Medium E supplemented with insulin, penicillin/streptomycin, L-glutamine, hydrocortisone, and heparin	SNMP (21°C)	ATP, AST, ALT, O_2_, bile production	ATP diminished during WI, higher ALT levels, and 1-month survival was 100%
		Group 2: 6		Group 2: 0						Bile production was 10 μl/g liver, 1-month survival was 83.3%
Tolboom et al. (34)	2012	Group 1: ≥4	Rat liver	1 h	–	Group 1: 5 h	Williams medium, autologous erythrocytes, insulin, heparin, penicillin/streptomycin, L-glutamine, hydrocortisone	Group 1: SNMP (20°C)	AST, ALT, bile secretion rate, the oxygen uptake rate	100% survival after 4 weeks
		Group 2: ≥4				Group 2: 5 h		Group 2: SNMP (30°C)		
Bruinsma et al. (32)	2014	7	Human liver	28 min	685 min	3 h	Williams’ Medium E supplemented with insulin, penicillin/streptomycin, hydrocortisone	SNMP (21°C)	Oxygen uptake, lactate, ATP, bile production, urea, albumin	Improved oxygen uptake and ATP content, increased bile production
Spetzler et al. (36)	2016	8	Porcine liver	–	3 h	3 h	Steen solution and washed pig erythrocytes	SNMP (33°C)	AST, ALP, bilirubin, histology	Perfusate AST level was in the normal range. Decreased AST and ALP levels after transplantation compared to CS. Bilirubin levels were constantly within the physiological range
*NMP*										
Vogel et al. (50)	2017	13	Human liver	3–15 min	280–964 min	24 h	Blood group compatible packed red blood cells and crystalloid solution. Total parenteral nutrition solution, heparin, bile salts, and prostacyclin were infused during perfusion	NMP	Bile production, lactate, oxygen consumption, histology	Bile production was significantly correlated with better histological grading, the level of necrosis seen in postperfusion biopsies was high
Nasralla et al. (48)	2018	121 (total 137, 16 livers discarded)	Human liver	21 min	–	11 h 54 min	Gelofusine and 3 units of donor-matched packed red blood cells. Antibiotics, heparin, insulin, prostacyclin, bile salts, and fat-free parenteral nutrition were infused during perfusion	NMP (37°C)	AST, bilirubin, lactate, INR, creatinine	Peak AST during the first week was reduced by 49.4%. Bilirubin level was lower compared to SCS. Graft survival after 1 year was 95%
Markmann et al. (44)	2022	151	Human liver	–	175.4 min	276.6 min	Buffered electrolyte solution, albumin, 4–5 units of packed red blood cells and supplemented with broad-spectrum antibiotics, continuous infusion of a nutrient solution of 4% amino acids and 10% dextrose, supplemented with insulin and multivitamins	NMP	Early allograft dysfunction (EAD), extend of reperfusion syndrome, incidence of ischemic biliary complications, post-transplant recipient survival	Decreased lactate levels, significant decrease in the incidence of EAD compared with ischemic cold storage group. One-month post-transplant survival rate was 99.3%
Clavien et al. (52)	2022	1	Human liver	–	1 h	First: 2 h 40 min	Packed red blood cells, fresh frozen plasma, thrombocyte concentrate, and human albumin 20%. Methylprednisolone, bicarbonate, heparin, parenteral nutrition, bile acid, caspofungin, meropenem, vancomycin, human insulin, glucagon, pheylephrine, and epoprostenol were infused	HMP (HOPE) (6°C–8°C)	AST, ALT, lactate, bile production, IL-6, factor V synthesis, histology	Fast clearance of lactate, decreased transaminases, low acute cytokine levels (IL-6) in the perfusate. Immediate histology after transplantation demonstrated a remarkable absence of inflammation with notably intact architecture, no necrosis and fully preserved intrahepatic bile ducts demonstrated 11 months after surgery
						Then: 68 h		NMP (37°C)		
*COR*										
Hoyer et al. (37)	2016	6	Porcine liver	–	18 h	3 h	Histidine tryptophan ketoglutarate preservation solution (Custodiol-N)	COR (from 8°C to 20°C)	AST, bile production, ATP, the activity of caspase 9	The activity of the mitochondrial caspase 9 was lower after COR. Measurement of tissue adenosine triphosphate and total adenine nucleotides at the end of the reconditioning period showed better energetic recovery
Furukori et al. (41)	2016	2	Porcine liver	60 min	4 h	4 h	UW solution	COR (from 4°C to 22°C)	AST, LDH, ATP, HA	The AST, LDH, and HA levels were lower compared with the HMP group
De Vries et al. (56)	2019	7 (5 of the livers were transplanted)	Human liver	23–35 min	289 min (median)	283–517 min (total machine perfusion time)	HBOC-201 supplemented with gelofusine, albumin, metronidazole, cefazolin, nutrients, glutathione, insulin, heparin, and NaHCO_3_	HMP (DHOPE)-COR-NMP	Lactate, bile production, ALT, oxygen consumption	100% graft survival at a median follow-up of 197 days, none of the recipients has developed clinical signs of post-transplant cholangiopathy

cfDNA, cell-free DNA; COR, controlled rewarming; CS, cold storage; DHOPE, dual hypothermic oxygenated machine perfusion; HA, hyaluronic acid; HMP, hypothermic machine perfusion; INR, international normalized ratio; NMP, normothermic machine perfusion; SCS, static cold storage; SNMP, subnormothermic machine perfusion; WI, warm ischemia.

### 3.1. Hypothermic machine perfusion

Notably, there are multiple liver perfusion protocols that employ hypothermic temperatures and oxygenation; for simplicity in presentation, we refer to all of these as HMP, with brief comments to their differences where needed.

HMP relies on decreased cellular metabolism at lower temperatures (4°C–10°C). HMP of the kidneys, but without oxygenation, has become standard practice in kidney perfusion, but with limited benefits compared to SCS (21). Guarrera et al. reported the first successful human liver transplantation after HMP (22), without active oxygenation. With HMP protocols featuring active oxygenation, studies have shown that despite lower temperatures, HMP helps organs to restore ATP levels (23), leading to reduced post-transplant complications (24). A similar study where DCD livers were treated with a short period of dual HMP (termed DHOPE) at the end of the ischemic period showed a reduced IRI of the biliary tree (25). Short-term (2 h) end-ischemic oxygenated HMP after SCS has also been shown to result in better endothelial cell function of ECD livers compared to SCS preservation alone (26). Clinical trials have shown superior transplantation outcomes of DCD livers after end-ischemic HMP (HOPE) compared to untreated DCD and DBD liver transplants: Schlegel et al. demonstrated higher 5-year graft survival (27), and Mueller et al. demonstrated reduced cancer recurrence in liver recipients who presented with hepatocellular cancer before the transplant (28). A key randomized controlled trial of HMP for DCD livers has shown a lower risk of non-anastomotic biliary strictures compared with SCS (6% in the machine-perfusion group vs. 18% in the control group) (29).

In terms of using HMP to extend the duration of preservation, one study tested HMP (DHOPE) for up to 24 h with success (30). The viability assessment at 24 h of HMP (DHOPE) was similar to the assessment of livers perfused for 2 and 6 h, and superior to 24-h cold-stored livers. Overall, these studies clearly demonstrated the utility of HMP and its superiority to SCS in most cases. The fact that HMP allowed ATP recovery of the grafts was somewhat surprising given the low temperature, where mitochondrial activity was assumed to be minimal at best. Indeed, many investigators explored a selection of warmer temperatures to enable improved mitochondrial function.

### 3.2. Subnormothermic machine perfusion

SNMP offers a balance between reducing the metabolic needs of the tissue and allowing the mitochondria to work and replenish ATP stores (31, 32). Another reason behind the development of SNMP has been operational simplicity. At normothermic temperatures, an oxygen carrier is required to meet the oxygen demands of the tissue, whereas at 21°C, supraphysiological levels of oxygenation suffice (33). Further, SNMP eliminates the need for a secondary dialysis circuit that is necessary for NMP to provide additional nutritional supply and removal of liver by-products (34). Multiple studies using animal or human liver models suggest that SNMP can positively affect liver viability and predict post-transplantation graft function (35–37). SNMP has also been shown to be equal to NMP in terms of allowing resuscitation of 1-h warm ischemic rat livers to transplantability (34, 38). A more thorough metabolomic analysis has shown that SNMP is superior in terms of faster recovery of ATP levels to nominal compared to NMP, but at the cost of somewhat increased oxidative stress, as indicated by depletion of glutathione and its precursors (39, 40).

NMP has specifically been tested to extend the preservation duration in an end-cold-storage mode (12), where it was shown to allow the doubling of total preservation time for rat livers (to 48 h) with 100% transplant success. SNMP of discarded human livers after a duration of cold ischemia resulted in remarkable improvement in liver viability factors (32). Another SNMP study demonstrated lower AST, LDH, and HA levels than HMP controls in perfused porcine livers (41).

### 3.3. Normothermic machine perfusion

At the most basic level, NMP aims to mimic physiological conditions precisely by maintaining body temperature (36°C–38°C), providing essential substrates for cellular metabolism—oxygen and nutrition (42)—and avoiding the cold ischemia, which, for marginal grafts, leads to an increased risk of graft failure and post-transplant mortality (43, 44). An additional benefit is that the organ is fully functional, and various markers can be readily measured and easily compared to *in vivo* function levels to gauge graft viability before transplantation (45, 46). NMP also allows pharmacological options on grafts to enhance graft repair (47), whereas, with HMP and SNMP, the reduced activity may render such pharmacologic compounds inactive. The first key randomized controlled trial demonstrated successful 12 h NMP for DCD livers: NMP DCD livers showed superior outcome data compared to DCD and DBD livers preserved with SCS (48). In another recent multicenter randomized controlled clinical trial, livers were preserved by either ischemic cold storage or NMP; the results showed remarkably better clinical outcomes and a higher use of DCD livers for transplantation in the NMP group (51% in the NMP group vs. 26% in the SCS group) (44).

NMP itself has shown significant potential in extending preservation durations. Even in the first clinical trials, the participating surgeons indicated they felt comfortable leaving the graft on the pump for a few more hours to better plan the surgeries (48). Studies have shown human livers successfully preserved at normal metabolic rates for 24 h (49, 50), with one preclinical study showing maintenance in a functional state for up to 1 week as assessed by bile production, ATP levels, and synthesis of blood proteins (51). A recent single clinical case by Clavien et al. showed long-term NMP for 68 h with successful transplantation, three times longer than in previous studies (52).

The concerns with NMP are that, at 37°C, the grafts are going at “full speed”, and, as sophisticated as an NMP system is, it does not capture and recapitulate the entire human body. Therefore, one concern is that extended NMP may exacerbate any shortcomings of the perfusion system. Other concerns include cost and lack of failsafe options should a pump failure occur, which has been one motivating factor behind controlled rewarming (COR).

### 3.4. Controlled rewarming and mixed perfusion modalities

Given that each temperature seems to have its advantages and drawbacks, one alternative approach is to use a combination therapy to prepare the graft more gently for transplant. COR aims to achieve this by starting the perfusion with SCS or HMP and linearly increasing the temperature to NMP. A key advantage is that during the initial cold preservation, a more straightforward protocol and the use of a device that is likely less costly and effort-intensive and minimizes the risk of technical issues with the pump. Moreover, should the pump fail, the fallback is SCS, which will occur in the same container without any user intervention.

Assuming everything goes as expected during the cold period, where the transportation of the graft to the recipient site is completed, the temperature is gently increased, presumably striking a balance similar to SNMP, but ramping up to NMP, where the graft can be easily assessed and is prepared for function *in vivo*. Indeed, better hepatic functions and increased bile production are achieved with a period of COR after the initial HMP (53). In one study, COR was compared with NMP for the resuscitation of liver grafts after cold storage. COR resulted in better energetic recovery and a higher bile production (37). Hoyer et al. demonstrated successful transplantation of human livers after COR with a 100% post-transplant survival rate at 1 and 3 years and a 93.8% survival rate at 5 years (54).

A similar approach is to sequence HMP and NMP. Treating DCD livers with HMP (DHOPE) before NMP has shown excellent results in several studies (23, 55). Using sequential HMP (DHOPE) and NMP, Porte et al. reported 100% graft survival after 197 days of follow-up without any post-transplant biliary complications (56). This protocol also increased the number of transplantable livers from rejected high-risk livers by 20% (57).

To our knowledge, COR or sequenced modalities have not been directly tested to extend preservation. However, sequencing static storage with a warm perfusion to recover from the cold and prepare the graft for transplantation has been the key pillar of subzero organ storage, which specifically aims to extend preservation durations.

## 4. Subzero organ preservation

Cryopreservation is the use of low temperatures, traditionally defined as −80°C (solid carbon dioxide) or −196°C (liquid nitrogen), to preserve structurally intact biological systems for periods in the order of years (58). It is an enabling technology with many medical applications, such as bone marrow transplantation, blood transfusion, artificial insemination, and *in vitro* fertilization (59).

When it comes to applying traditional cryopreservation to vital organs, there are a variety of challenges that the field has struggled with for decades. When large (up to 2–3 L of volume in the case of the liver) and three-dimensional structures are cooled below freezing point, ice can immediately form (60). This ice directly damages cells, as intracellular formation can rupture cells and extracellular formation can cause severe mechanical stress (61). The standard approaches to mitigate cryoinjury include the following: (1) the use of cryoprotectants (e.g., DMSO, glycerol, sorbitol, 2,3-butanediol) that modulate the thermodynamic properties of the sample (freezing point depression, altering the type of ice that forms, dehydration of cells before freezing, etc.) (59); and (2) careful tuning of cooling and rewarming rates to allow some spatiotemporal control of ice formation (62). Cooling too fast can cause insufficient dehydration of cells and increases the risk of lethal ice formation (63), whereas cooling too slowly means an extended period of ischemic injury. Similarly, for the re-warming stage, rapid thawing at 37°C–40°C is considered crucial (64) to prevent ice reformation and other mechanisms of damage to the cells. However, there have been few, if any, successes in scaling up traditional cryopreservation—which we define as any method that involves allowing ice formation in the range of −80°C to −196°C.

Recent research efforts have taken very different paths, either avoiding ice altogether or aiming to achieve temporal control using novel nature-inspired techniques. For the purposes of clarity in presentation, we will delineate the approaches into two categories (Figure 2): high subzero, meaning the range of −4°C to −30°C and covering supercooling, isochoric, and partial freezing; and low subzero storage, meaning vitrification at −196°C, particularly involving nanowarming. Table 2 depicts key developments in this new field. Table 3, in turn, provides an extended summary of all key extended preservation studies, including both subzero preservation and machine perfusion. It should be noted that the technological readiness level of the approaches described here are at a much earlier stage compared to machine perfusion overall, with only one of them having been scaled to human livers already. In a few cases, the initial development efforts have focused on kidneys, but we have opted to include them in this discussion as efforts in translation to livers have already been described.

**Table 2:** Novel subzero approaches in organ preservation.

	Low subzero			High subzero		
	Slow freezing	Vitrification	Nanowarming	Supercooling	partial freezing	Isochoric subzero
Application	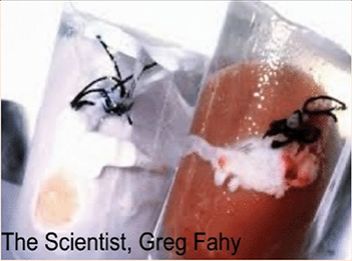 (84)		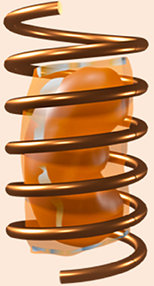 (93)	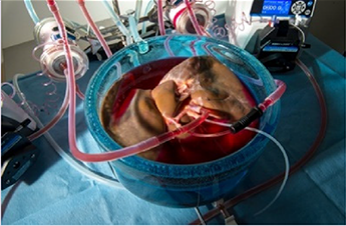 (71)	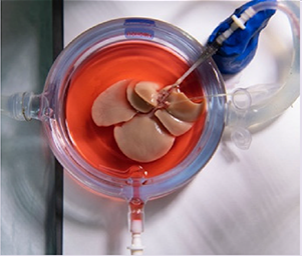	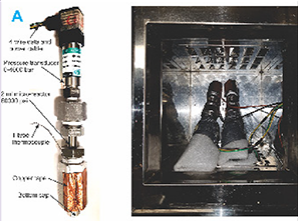 (80)
Description	Storage below freezing point and ice formation	Rapid cooling into glass phase without ice formation	Rapid and uniform rewarming with nanoparticles without ice	Storage below freezing point without ice formation	Storage below freezing point with controlled ice	Constant volume ice-free storage
Key component	CPA mix	CPA mix	SPIONs and CPA mix	3-OMG and PEG	PEG, 3-OMG, Snomax	Constant volume
Temperature range	−120°C to −196°C			−4°C to −6°C	−10°C to −15°C	−5°C to −30°C
Thermodynamic state	Equilibrium	Non-equilibrium	Technically non-equilibrium but for all purposes of transplant stable	Non-equilibrium	Equilibrium	Equilibrium
Storage time	Years			Days to weeks	Weeks to months	
Main problem	Ice damage	CPA toxicity, devitrification	Technically challenging	Unstable, limited storage size	Ice damage	Technically challenging

CPA, cryoprotective agents.

**Table 3:** A brief summary of key studies in extended organ preservation.

Author	Year	Organ type	Main protective agents/strategy	Recovery MP		Storage time	Perfusate	Temperature	Endpoints	Outcome
*Machine perfusion*										
Clavien et al. (52)	2022	Human liver	Oxygenated perfusion	NMP		68 h	Packed red blood cells, fresh frozen plasma, thrombocyte concentrate, and human albumin 20%. Methylprednisolone, bicarbonate, heparin, parenteral nutrition, bile acid, caspofungin, meropenem, vancomycin, human insulin, glucagon, pheylephrine, and epoprostenol were infused	37°C	AST, ALT, lactate, bile production, IL-6, factor V synthesis, histology	Fast clearance of lactate, decreased transaminases, low acute cytokine levels (IL-6) in the perfusate. Immediate histology after transplantation demonstrated a remarkable absence of inflammation with notably intact architecture, no necrosis and fully preserved intrahepatic bile ducts demonstrated 11 months after surgery
Bruggenwirth et al. (30)	2020	Porcine liver	Oxygenated perfusion	HMP (DHOPE)		24 h	Belzer UW machine perfusion solution oxygenated with 100% O_2_		Lactate clearance, glucose, ALT, LDH, HMGB-1, IL-6, TNFα, cfDNA, bile duct, and liver histology	All livers cleared lactate during HMP (DHOPE). Histological analysis of liver parenchyma revealed that HMP (DHOPE) for up to 24 h did not result in more necrosis after reperfusion compared to shorter preservation times. Extended HMP (DHOPE) was not associated with increased release of inflammatory cytokines TNFα and IL-6
*Supercooling*										
Berendsen et al. (69)	2014	Rat liver	PEG, 3-OMG	Loading: 1 h SNMP Unloading: 3 h SNMP		72–96 h	Williams’ Medium E, L-glutamine, penicillin/streptomycin hydrocortisone, heparin, insulin with 3-OMG	−6°C	3-month survival rate, postoperative albumin, ALP, urea, coagulation times	Three-month survival was 58% after 96 h of preservation, and 100% after 72 h of preservation. Postoperative blood levels of albumin, bilirubin, alkaline phosphatase, and blood urea normalized within 1 month postoperatively, and coagulation times were normal in all animals
De Vries et al. (71)	2019	Human liver	PEG, 3-OMG	Loading: 3 h SNMP, 1 h HMP Unloading: 10 min HMP, 3 h SNMP		20 h	Perfusate supported with 3-OMG for SNMP	−4°C	Bile production, lactate, AST, ALT	Three livers produced the same amount of bile during SNMP before and after supercooling, indicating successful preservation
*Isochoric subzero*										
Wan et al. (82)	2018	Rat heart	Constant volume	Unloading: Langendorff perfusion at 37°C		1 h	UW solution	−4°C, −6°C, −8°C	The rate of contraction (beats per minute) and flow rate (ml/min)	Hearts preserved at −6°C suffered injury from cellular swelling and extensive edema, and at −8°C hearts experienced significant morphological disruption. Tissue damage was observed at lower temperatures (−6°C and below)
*Partial freezing*										
Tessier et al. (79)	2022	Rat liver	3-OMG, PEG, glycerol	Loading: 80 min SNMP, 45 min HMP Unloading: 60 min HMP, 180 min SNMP		5 days	William's medium E, 3-OMG	−10°C to −15°C	pH, lactate, bile, O_2_, AST, ALT, LDH, cfDNA, TNFα, albumin, IL-8	All livers produced bile. pH decreased throughout perfusion. Mean ALT 768 ± 206 U/L. Mean AST 1,270 ± 430 U/L
*Vitrification*										
Fahy et al. (84)	1984	Rabbit kidney	VS55	Ramp loading		NA	DF_10_ + 6% PVP in RPS-2 (renal perfusion solution-2)	−145°C	Vitrification	Ice free vitrification (no rewarming)
Fahy et al. (103)	2009	Rabbit kidney	M22	Ramp loading		8 min	M22 in LM5	−130°C	Histology transplant with measurement of Cr, K+, and hemoglobin	First: 9-day survival with partial function Second: 50-day survival with partial function (nadir Cr ∼4)
*Nanowarming*										
Sharma et al. (93)	2021	Rat kidney	VS55	Step loading	Step Unload	Not reported	VS55 in Euro-Collins	−150°C	Micro CT, histology	Ice-free vitrification, preserved tissue morphology and intact endothelium
Chiu-Lam et al. (95)	2021	Rat heart	VS55	Step loading	Step Unload	1 week	VS55 in Euro-Collins	−196°C	Nanoparticle distribution by Prussian blue histology and CT imaging	Successful loading and unloading (90%) of SPIONs, no gross macro histologic changes
Gao et al. (94)	2022	Rat heart	VS55	Step loading	Step Unload	Not reported	VS55 in Euro-Collins	−150°C	MicroCT, histology, electrical activity	Ice-free vitrification, some cardiac electrical activity, histologically similar to CPA-only treated organs

cfDNA, cell-free DNA; CPA, cryoprotective agents; DHOPE, dual hypothermic oxygenated machine perfusion; HMP, hypothermic machine perfusion; MP, machine perfusion; NA, not applicable; NMP, normothermic machine perfusion; SNMP, normothermic machine perfusion; SPION, superparamagnetic iron oxide nanoparticles; UW, University of Wisconsin.

### 4.1. High subzero storage

#### 4.1.1. Supercooling

Ice nucleation is a stochastic event; under atmospheric pressure, the probability of ice nucleation becomes nonzero at 0°C and increases as the temperature is reduced. This means it is possible for biological systems to stay below the freezing point, which is called supercooling. Contrary to expectations, supercooling just below the freezing point can be remarkably stable. Indeed, supercooling is employed as a preservation technique by multiple animals in nature, including one crocodile. Still, the poster child is the arctic ground squirrel (65), which is known to have core temperatures of −8°C in a supercooled state. Given that every 10°C drop in temperature roughly halves the metabolic rates, a doubling of preservation time is relatively easy by reducing the storage temperature from ice-cold (2°C–8°C effective in the organ depending on the point of measurement) to a relatively easy to attain −5°C, while avoiding the complications and injuries induced by phase change (66).

The basic idea of utilizing supercooling in the transplantation field goes back at least to the 1960s (67). However, it appears to have been abandoned in the transplantation field since the advent of SCS. Recent developments, however, demonstrated that supercooling improved the preservation of primary hepatocytes (68), which was then scaled to rat livers with this success (69): The investigators demonstrated that SCS allowed only 24 h of storage of rat livers with transplant success, with 100% survival at 30 days. At 2 days, success fell to only 50%; at 3 days or longer, there was no survival. Machine perfusion alone was shown to double preservation to 2 days with excellent success but failed to push beyond (0% survival at 3 days) (12). The tandem use of subzero storage at −6°C, machine perfusion (SNMP), and two select preservative agents 3-*O*-methyl glucose (3-OMG) (70) and *polyethylene glycol* (PEG) have allowed transplantations with success at 3 days, with 4 days supercooled storage possible but a reduction in survival (69). The technique was then scaled up to whole human livers in a preclinical study, with success demonstrated when five human livers were preserved ice-free for 27 h at −4°C and followed by SNMP (71, 72). Given that organs only rejected for transplant are available for research, viability was tested using a matched non-inferiority trial comparing the state of the livers before and after as assessed during SNMP, followed by a simulated transplant step including reperfusion with reconstituted whole blood. The key changes in the human protocol are additional cryoprotective agents (CPAs) of glycerol and trehalose to enable stable, non-frozen storage and a multi-temperature perfusion protocol to enable homogenous loading of CPAs and their safe removal before rewarming (72).

As supercooling is not an asymptotically stable state by thermodynamic standards, it is often assumed that there is a significant risk of ice formation in the organ over the durations of storage practical for transplantation. In an elegant study, Huang et al. demonstrated that the risk of freezing is effectively zero so long as the heterogenous nucleation is prevented by eliminating air/liquid interfaces that induce ice formation (73). In this work, the air/water interface was eliminated using an oil sealant such that a large volume of water (100 ml) can be kept at −16°C for up to 100 days without freezing, and, similarly, red blood cells in 30-ml suspensions can be readily preserved at −13°C for up to 365 days. The same approach has also succeeded in preserving adipose-derived stem cells at −13°C and −16°C (74). This approach, dubbed deep supercooling, comes close to eliminating heterogeneous nucleation and leaves only homogenous nucleation as a possible risk for ice formation. Notably, the homogenous freezing temperature of water, i.e., the point where it becomes thermodynamically feasible for H_2_O molecules to form crystals in the absence of a catalyst such as an air–liquid interface, is −20°C (75), which therefore is the actual thermodynamic limit of supercooled storage.

Of the subzero approaches, supercooling is the most advanced in terms of technological readiness, with success in preclinical human studies already demonstrated. The key advantage it possesses is the absence of a need to deal with the consequences of phase change, be it glass or ice. Similarly, the CPAs used are very benign and are required in low concentrations compared to many alternatives. In terms of equipment, it is equivalent to a standard perfusion pump plus one chiller, which is technically not difficult to combine. All these advantages make it of higher value from a translational perspective, but avoiding phase change also limits the attainable preservation duration.

#### 4.1.2. Partial freezing

While the homogenous nucleation temperature thus limits supercooling, many organisms in nature can handle ice formation and use it as a method of survival over harsh winters. The classic example of this approach is the wood frog, *Rana Sylvatica* (76, 77), which is known to survive over 200 days in a frozen state in lab conditions. A key aspect is that the ice does not occur randomly, but its formation is carefully controlled both spatially and temporally; the result is that the animal's blood is completely frozen, but the parenchyma remains in a non-frozen subzero state similar to supercooling. In a study using a microfluidic model of capillaries, a similar spatiotemporal control was first shown to be possible using Snomax, a well-known ice-inducer that is used to create artificial snow for ski resorts (78). Leveraging these findings, the same group demonstrated the successful partial freezing of rodent livers for 5 days at temperatures as low as −15°C (79). The study further demonstrated that the main limitation was the freezing-thawing process, and it may be possible to extend the storage duration further to reach that achieved by *Rana Sylvatica*. Extrapolating from the numbers observed in nature, partial freezing has the potential to further push the limits of high subzero preservation from weeks to months. However, the phase change still causes some injury to the organs, as suggested by elevated ALT levels, and further improvements are needed to scale to human organs.

#### 4.1.3. Isochoric preservation

Isochoric (constant volume) preservation is a novel approach aiming to utilize pressure as an additional variable to optimize preservation conditions. The process involves placing the biological sample suspended in preservation media within a pressure chamber and inducing ice formation at the periphery of the chamber (80). Since the molar volume of ice is larger than liquid water, the ice expands and increases the pressure of the liquid trapped in the chamber, which depresses the freezing point for the remaining liquid (specimen) and allows storage at lower temperatures in a non-frozen state without the need of additional, potentially toxic, CPAs (81). Initial studies with rat hearts have shown preservation under isochoric conditions at −8°C (78 MPa) in a UW solution for 1 h without additional CPAs (82). And in cardiac micro physiological systems, the use of isochoric conditions has been shown to increase the stability of supercooling by preventing the system to access air-water interfaces and suppressing density fluctuations (83). While there is much work to be done in terms of scaling towards transplantation, and success has not yet been shown in models of liver transplantation, the fundamental idea of controlling pressure and temperature makes intuitive and thermodynamic sense and can enable entirely new applications and technologies.

### 4.2. Low subzero storage

#### 4.2.1. Vitrification

Under cryogenic conditions (defined as temperatures <−153°C), metabolic activity effectively ceases, and the storage of biologic material is theoretically indefinite (84). The application of this ultralow temperature storage of biomaterials was conceptualized by Boyle in the 17th century when trying to preserve human bodies and animals (85). Still, it was not successfully achieved until the 1940s when Polge, Smith, and Parkes demonstrated that the addition of CPAs, such as glycerol, could protect cells at ultralow temperatures and enable the successful cryopreservation and recovery of spermatozoa (86). Unfortunately, those conventional cryopreservation techniques failed with larger specimens, such as tissues and organs, due to the destructive formation of ice crystals that kills cells (87) and disrupts the complex architecture of whole organs (88, 89).

Vitrification, or rapid cooling to a glass-like state, offers a promising alternative to conventional cryopreservation (88). The biomaterial is cooled at a rate that is too fast for the phase transition from liquid to crystalline ice to occur. Basel Luyet is generally credited with conceptualizing this approach in the 1930s (90), but the first successful demonstration occurred with erythrocytes in the 1960s (91). In the vitrified state, the biomaterial is an amorphous solid and can be cryogenically stored without any destructive ice formation. The first major biological and physical success of vitrification and rewarming was achieved with mouse embryos in the 1980s (92), thereby opening up the field of assisted reproductive medicine. The extension of vitrification to organs was shown by Fahy and colleagues in the mid-1980s, when they showed that rabbit kidneys that had been perfused with sufficiently concentrated CPA solutions could be cooled to an ice-free vitrified state. Vitrification of other organs, including rat kidneys (93), hearts (94, 95), livers (96), porcine blood vessels (97), and ovaries (98), has also been demonstrated more recently.

However, rewarming vitrified organs while preserving viability and function remains challenging. To rewarm them, the rate of warming must be fast enough to avoid ice formation during warming. That rate, the critical warming rate (CWR), is always greater than the critical cooling rate (CCR; the rate to achieve vitrification), which is already incredibly high for physiologic solutions (>10^7^°C/min) which cannot be reached for any but the smallest size specimens (99). Fortunately, CPA cocktails have been developed that reduce the CWR to achievable values (100, 101). However, these CPAs can be toxic to cells and tissues. Thus, there is a critical balance between achieving sufficient CPA concentration in tissue and avoiding the toxicity of those agents. The equilibration of CPA in the tissue or organ increases the CPA exposure time, which can be toxic to cells (102). Combining multiple CPAs with similar thermodynamic effects but different underlying toxicity mechanisms, or lowering the CPA exposure temperature, are options commonly pursued to reduce the toxicity.

Further, the rewarming of organs must be uniform. Boundary heating, such as convection during immersion in a water bath, fails in organs and other larger biomaterials. This is because the temperature gradient from the warmed surface to the relatively cooler core creates significant thermal stresses that can fracture the organ system. Thus, successful rewarming of vitrified organs must be fast (to avoid ice) and uniform (to prevent cracking or fracture)—two challenges that are difficult to achieve in combination.

#### 4.2.2. Nanowarming

Several approaches to rewarming vitrified organs have been attempted, including convective warming (103), dielectric heating (104), and even microwave heating (105–107). However, these have yet to achieve sufficiently rapid and uniform heating to restore full function in samples greater than several milliliters in size. To overcome these limits, a new electromagnetic approach called “nanowarming” was developed (108) and deployed on an 80-ml system (97). It was then generalized to the kidneys (and later other organs) that could be perfused with CPA and biocompatible iron oxide nanoparticles (109) and then heated (93). The CPA diffuses throughout the tissue, but the nanoparticles remain intravascular. The organs are then rapidly cooled to a vitrified state and stored at an ultralow temperature (i.e., −150°C) until needed. To rewarm them, the organs are transferred to a radiofrequency (RF) coil that induces heat generation through hysteresis losses from oscillating magnetic dipoles in the nanoparticles. Rewarming is both rapid and uniform.

Since RF waves penetrate biomaterials efficiently, and the nanoparticles are distributed throughout the vasculature, the process of nanowarming is essentially independent of system size and can scale from rodent to human organs. The physical success of this approach has been demonstrated for the blood vessels, heart, kidney, and liver in animal models (93–97). However, future work will be needed to minimize CPA toxicity for improved organ function and scaling various aspects of the protocols to human organ size.

## 5. Discussion

In many aspects, transplantation is a story of moving from one extreme to another. The starkest contrast is the extremes found between death due to organ failure and a decade or more of life a transplant can provide. The immunosuppression required enables transplant but increases the risk of cancer and infection. The preservation methods have oscillated between warm and cold and warm again, and as this review of recent developments indicates, the next-generation techniques are evidently returning to cold.

Prolonged *ex vivo* machine liver perfusion has been explored clinically, with one major study showing success with up to 3 days (52), and prior clinical experience also suggests using it to optimize scheduling. A storage time of 3 days itself is a major improvement, which could transform the current clinical practice by making transplant an elective surgery. As to the type of perfusion modality, it is difficult to suggest the best approach, since there are very few detailed comparative studies. Each temperature has its advantages and challenges; NMP keeps the livers fully functional and allows the most straightforward way for viability assessment before transplant. The low temperature of HMP provides low metabolic rates with decreasing cellular metabolic need. SNMP, as well as COR, offer a balance between these two approaches, and there appears to be some evidence suggesting that a gentler approach may be the ideal option. Rigorous studies comparing these alternatives, ideally in a clinically relevant large animal model, could answer many key questions and inform the field. On the other hand, it is unclear who would fund such a comparative study. The end result may be that each transplant center will have its preferred protocol, likely based on the cost aspects, and the comparison will have to wait until there are enough data to perform a retrospective study.

For the subzero approaches in development, the literature indicates there is a variety of techniques in development, all with promising results, with different strengths and weaknesses in terms of potential storage duration vs. complexity. The likely near-term trajectory is that these techniques will be developed towards clinical testing in order of technological readiness levels. It is possible to envision a future where there are multiple preservation techniques employed based on need: for ideal organs with a recipient in close proximity, SCS can be perfectly adequate; for organs that need repair, some form of perfusion would allow *ex vivo* treatment, which would further allow allocation of organs in an expanded area such as within the continental USA. For international exchanges and allocation and short-term banking in the order of months, for instance to provide back-up livers to be used in case of Primary Nonfunction (PNF) and acute liver injury, high subzero organ banking would be the solution. For very long-term banking, which would allow off-the-shelf readiness for organs, low subzero approaches would be the real solution. Isochoric approaches could either boost one of these or be completely enabling by, for instance, allowing a specific approach to be practical without the need for using toxic levels of CPA. Whether these options can simultaneously be viable commercially will depend on cost, practicality, and efficacy, which are yet to be determined.

For the clinical translation of the novel techniques described here, the economics of the technology are a key aspect of widespread adoption. Cold storage is simple and cheap compared to the cost of the graft, not to mention the total cost of the transplant procedure, including postoperative patient care. The latter component, the cost of patient care, can more than double the cost of the graft and surgical procedures, since it involves intensive care, extending significantly depending on the outcomes. When these costs are included, machine perfusion is cost-effective considering the significantly decreased rates of transplant complications, reduced length of hospital stay, and consequent benefits to the transplant waiting list (110). It can be readily envisioned that similar benefits will also be true for new techniques for organ preservation. It is too early to make a cost comparison between various subzero approaches. However, the financial structure of how those costs are paid and how risk is balanced between the organ procurement organization, transplant centers, and insurers must be determined. These structures are also likely to vary between countries with different laws, healthcare systems, and ethical and cultural views on organ transplantation. Technology, as it often does, will likely push a closer alignment of such structures globally.

The most extreme aspect of new biopreservation technologies will be their impact on our unique system for allocating scarce but lifesaving resources. The existing organ availability and allocation system is predicated upon an organ's time-limited viability when outside the body. However, as technology modifies that barrier, the system must change. Significantly prolonging *ex vivo* viability would permit greater flexibility with candidate identification for a transplant and better candidate preparation, permitting safer transplant procedures. There will also be “new” variables entered into the assessment of organ quality. Will medical judgment continue as the final tool to assess organ quality? Introducing complex physical and chemical organ manipulation will likely exceed clinical expertise. Oversight, required to assure public trust, will be challenged with expanded definitions for organ quality and safety. Questions about allocating organs equitably and ethically will become more prominent, and more complicated because it is rapidly becoming practical to share donor organs across countries, if not globally. The legal and ethical questions raised by the prospect of sharing organs without borders can be daunting to the extreme for some, but new allocation systems enabled by exciting new technology carry the promise of increasing access to this unique, lifesaving treatment, and a second transplant revolution.

It should be obvious that this work has its limitations. While extending the review to organs beyond the liver would have been preferrable in terms of completeness, we realized the scope grew beyond tractability, and we therefore refrained from discussing other organs with very few exceptions where translational technology transfer was obvious and provided insights. We similarly limited the scope of the review to isolated organs: NRP (111) and ECMO (112) are technologies that provide options to retain multiple organs *in situ* in the donor body, in a viable state for procurement at an ideal point in time. These techniques are exciting new options that would also enable addressing the unmet need for transplants and deserve their own separate review and discussion.

## 6. Conclusions

Recent studies have demonstrated that a variety of new strategies in preservation promises to enable longer storage times and recover marginal donor organs. Machine perfusion has the potential to be the clinical standard for transplant and is evolving rapidly before our eyes. Tomorrow will belong to next-generation preservation techniques, which may be advanced perfusion or subzero or isochoric, or something else that is brewing on a lab bench. What is clear is that transplantation is seeing the convergence of several new groundbreaking technologies, and the future will be very different from what is the current clinical standard across the world.

## References

[1] Giwa S Lewis JK Alvarez L Langer R Roth AE Church GM The promise of organ and tissue preservation to transform medicine. Nat Biotechnol. (2017) 35(6):530–42. 10.1038/nbt.3889 28591112 PMC5724041

[2] A fairer and more equitable, cost-effective, and transparent system of donor organ procurement, allocation, and distribution. Available at: https://www.nationalacademies.org/our-work/a-fairer-and-more-equitable-cost-effective-and-transparent-system-of-donor-organ-procurement-allocation-and-distribution.

[3] OPTN/SRTR 2018 annual data report: introduction. Am J Transplant. (2020) 20(Suppl s1):11–9. 10.1111/ajt.15671 31898409

[4] Tan SY Merchant J. Joseph Murray (1919–2012): first transplant surgeon. Singapore Med J. (2019) 60(4):162–3. 10.11622/smedj.2019032 31069396 PMC6482420

[5] Starzl TE Groth CG Brettschneider L Penn I Fulginiti VA Moon JB Orthotopic homotransplantation of the human liver. Ann Surg. (1968) 168(3):392–415. 10.1097/00000658-196809000-00009 4877589 PMC1387344

[6] Southard JH Belzer FO. Organ preservation. Annu Rev Med. (1995) 46:235–47. 10.1146/annurev.med.46.1.235 7598460

[7] Belzer FO Ashby BS Gulyassy PF Powell M. Successful seventeen-hour preservation and transplantation of human-cadaver kidney. N Engl J Med. (1968) 278(11):608–10. 10.1056/NEJM196803142781108 4866541

[8] Collins GM Bravo-Shugarman M Terasaki PI. Kidney preservation for transportation. Initial perfusion and 30 h’ ice storage. Lancet. (1969) 2(7632):1219–22. 10.1016/S0140-6736(69)90753-3 4187813

[9] Wahlberg JA Southard JH Belzer FO. Development of a cold storage solution for pancreas preservation. Cryobiology. (1986) 23(6):477–82. 10.1016/0011-2240(86)90056-8 3802886

[10] Salehi S Tran K Grayson WL. Advances in perfusion systems for solid organ preservation. Yale J Biol Med. (2018) 91(3):301–12. 30258317 PMC6153619

[11] Weissenbacher A Vrakas G Nasralla D Ceresa CDL. The future of organ perfusion and re-conditioning. Transpl Int. (2019) 32(6):586–97. 10.1111/tri.13441 30980772 PMC6850430

[12] Bruinsma BG Berendsen TA Izamis ML Yarmush ML Uygun K. Determination and extension of the limits to static cold storage using subnormothermic machine perfusion. Int J Artif Organs. (2013) 36(11):775–80. 10.5301/ijao.5000250 24338652 PMC4091033

[13] Dubbeld J Hoekstra H Farid W Ringers J Porte RJ Metselaar HJ Similar liver transplantation survival with selected cardiac death donors and brain death donors. Br J Surg. (2010) 97(5):744–53. 10.1002/bjs.7043 20393979

[14] Fernandez AR Sanchez-Tarjuelo R Cravedi P Ochando J Lopez-Hoyos M. Review: ischemia reperfusion injury – a translational perspective in organ transplantation. Int J Mol Sci. (2020) 21(22). 10.3390/ijms21228549 PMC7696417 33202744

[15] Perico N Cattaneo D Sayegh MH Remuzzi G. Delayed graft function in kidney transplantation. Lancet. (2004) 364(9447):1814–27. 10.1016/S0140-6736(04)17406-0 15541456

[16] Raigani S De Vries RJ Carroll C Chen YW Chang DC Shroff SG. Viability testing of discarded livers with normothermic machine perfusion: alleviating the organ shortage outweighs the cost. Clin Transplant. (2020) 34(11):e14069. 10.1111/ctr.14069 32860634 PMC7944462

[17] Resch T Cardini B Oberhuber RA Weissenbacher A Dumfarth J Krapf C Transplanting marginal organs in the era of modern machine perfusion and advanced organ monitoring. Front Immunol. (2020) 11:631. 10.3389/fimmu.2020.00631 32477321 PMC7235363

[18] Bruinsma BG Avruch JH Sridharan GV Weeder PD Jacobs L Crisalli K Peritransplant energy changes and their correlation to outcome after human liver transplantation. Transplantation. (2017) 101(7):1637–44. 10.1097/TP.0000000000001699 28230641 PMC5481470

[19] Agius T Songeon J Klauser A Allagnat F Longchamp G Ruttimann R Subnormothermic ex vivo porcine kidney perfusion improves energy metabolism: analysis using (31)P magnetic resonance spectroscopic imaging. Transplant Direct. (2022) 8(10):e1354. 10.1097/TXD.0000000000001354 36176724 PMC9514833

[20] Karangwa SA Dutkowski P Fontes P Friend PJ Guarrera JV Markmann JF Machine perfusion of donor livers for transplantation: a proposal for standardized nomenclature and reporting guidelines. Am J Transplant. (2016) 16(10):2932–42. 10.1111/ajt.13843 27129409 PMC5132023

[21] Moers C Smits JM Maathuis MH Treckmann J van Gelder F Napieralski BP Machine perfusion or cold storage in deceased-donor kidney transplantation. N Engl J Med. (2009) 360(1):7–19. 10.1056/NEJMoa0802289 19118301

[22] Guarrera JV Henry SD Samstein B Odeh-Ramadan R Kinkhabwala M Goldstein MJ Hypothermic machine preservation in human liver transplantation: the first clinical series. Am J Transplant. (2010) 10(2):372–81. 10.1111/j.1600-6143.2009.02932.x 19958323

[23] van Rijn R Karimian N Matton APM Burlage LC Westerkamp AC van den Berg AP Dual hypothermic oxygenated machine perfusion in liver transplants donated after circulatory death. Br J Surg. (2017) 104(7):907–17. 10.1002/bjs.10515 28394402 PMC5484999

[24] Schlegel A Porte R Dutkowski P. Protective mechanisms and current clinical evidence of hypothermic oxygenated machine perfusion (HOPE) in preventing post-transplant cholangiopathy. J Hepatol. (2022) 76(6):1330–47. 10.1016/j.jhep.2022.01.024 35589254

[25] van Rijn R van Leeuwen OB Matton APM Burlage LC Wiersema-Buist J van den Heuvel MC Hypothermic oxygenated machine perfusion reduces bile duct reperfusion injury after transplantation of donation after circulatory death livers. Liver Transpl. (2018) 24(5):655–64. 10.1002/lt.25023 29369470 PMC5947530

[26] Burlage LC Karimian N Westerkamp AC Visser N Matton APM van Rijn R Oxygenated hypothermic machine perfusion after static cold storage improves endothelial function of extended criteria donor livers. HPB. (2017) 19(6):538–46. 10.1016/j.hpb.2017.02.439 28351756

[27] Schlegel A Muller X Kalisvaart M Muellhaupt B Perera M Isaac JR Outcomes of DCD liver transplantation using organs treated by hypothermic oxygenated perfusion before implantation. J Hepatol. (2019) 70(1):50–7. 10.1016/j.jhep.2018.10.005 30342115

[28] Mueller M Kalisvaart M O'Rourke J Shetty S Parente A Muller X Hypothermic oxygenated liver perfusion (HOPE) prevents tumor recurrence in liver transplantation from donation after circulatory death. Ann Surg. (2020) 272(5):759–65. 10.1097/SLA.0000000000004258 32889870

[29] van Rijn R Schurink IJ de Vries Y van den Berg AP Cortes Cerisuelo M Darwish Murad S Hypothermic machine perfusion in liver transplantation: a randomized trial. N Engl J Med. (2021) 384(15):1391–401. 10.1056/NEJMoa2031532 33626248

[30] Bruggenwirth IMA van Leeuwen OB de Vries Y Bodewes SB Adelmeijer J Wiersema-Buist J Extended hypothermic oxygenated machine perfusion enables ex situ preservation of porcine livers for up to 24 h. JHEP Rep. (2020) 2(2):100092. 10.1016/j.jhepr.2020.100092 32195456 PMC7078381

[31] Bruinsma BG Sridharan GV Weeder PD Avruch JH Saeidi N Ozer S Metabolic profiling during ex vivo machine perfusion of the human liver. Sci Rep. (2016) 6:22415. 10.1038/srep22415 26935866 PMC4776101

[32] Bruinsma BG Yeh H Ozer S Martins PN Farmer A Wu W Subnormothermic machine perfusion for ex vivo preservation and recovery of the human liver for transplantation. Am J Transplant. (2014) 14(6):1400–9. 10.1111/ajt.12727 24758155 PMC4470578

[33] Bruinsma BG Avruch JH Weeder PD Sridharan GV Uygun BE Karimian NG Functional human liver preservation and recovery by means of subnormothermic machine perfusion. J Vis Exp. (2015) 98. 10.3791/52777 PMC4420550 25938299

[34] Tolboom H Izamis ML Sharma N Milwid JM Uygun B Berthiaume F Subnormothermic machine perfusion at both 20 degrees C and 30 degrees C recovers ischemic rat livers for successful transplantation. J Surg Res. (2012) 175(1):149–56. 10.1016/j.jss.2011.03.003 21550058 PMC3863393

[35] Fontes P Lopez R van der Plaats A Vodovotz Y Minervini M Scott V Liver preservation with machine perfusion and a newly developed cell-free oxygen carrier solution under subnormothermic conditions. Am J Transplant. (2015) 15(2):381–94. 10.1111/ajt.12991 25612645 PMC5024042

[36] Spetzler VN Goldaracena N Echiverri J Kaths JM Louis KS Adeyi OA Subnormothermic ex vivo liver perfusion is a safe alternative to cold static storage for preserving standard criteria grafts. Liver Transpl. (2016) 22(1):111–9. 10.1002/lt.24340 26390093

[37] Hoyer DP Paul A Luer S Reis H Efferz P Minor T. End-ischemic reconditioning of liver allografts: controlling the rewarming. Liver Transpl. (2016) 22(9):1223–30. 10.1002/lt.24515 27398813

[38] Berendsen TA Bruinsma BG Lee J D'Andrea V Liu Q Izamis ML A simplified subnormothermic machine perfusion system restores ischemically damaged liver grafts in a rat model of orthotopic liver transplantation. Transplant Res. (2012) 1(1):6. 10.1186/2047-1440-1-6 23369351 PMC3552573

[39] Karimian N Raigani S Huang V Nagpal S Hafiz EOA Beijert I Subnormothermic machine perfusion of steatotic livers results in increased energy charge at the cost of anti-oxidant capacity compared to normothermic perfusion. Metabolites. (2019) 9(11). 10.3390/metabo9110246 31652927 PMC6918199

[40] Zhang A Carroll C Raigani S Karimian N Huang V Nagpal S Therapeutic implications of amino acid metabolism in non-steatotic discarded human livers during normothermic versus subnormothermic machine perfusion. Hepatology. (2020) 72:854A–854A.

[41] Furukori M Matsuno N Meng LT Shonaka T Nishikawa Y Imai K Subnormothermic machine perfusion preservation with rewarming for donation after cardiac death liver grafts in pigs. Transplant Proc. (2016) 48(4):1239–43. 10.1016/j.transproceed.2015.12.076 27320595

[42] Reddy SP Brockmann J Friend PJ. Normothermic perfusion: a mini-review. Transplantation. (2009) 87(5):631–2. 10.1097/TP.0b013e3181995e83 19295304 PMC5842890

[43] Vogel T Brockmann JG Coussios C Friend PJ. The role of normothermic extracorporeal perfusion in minimizing ischemia reperfusion injury. Transplant Rev. (2012) 26(2):156–62. 10.1016/j.trre.2011.02.004 22459038

[44] Markmann JF Abouljoud MS Ghobrial NM Bhati CS Pelletier SJ Lu AD Impact of portable normothermic blood-based machine perfusion on outcomes of liver transplant: the OCS liver PROTECT randomized clinical trial. JAMA Surg. (2022) 157(3):189–98. 10.1001/jamasurg.2021.6781 34985503 PMC8733869

[45] Mergental H Stephenson BTF Laing RW Kirkham AJ Neil DAH Wallace LL Development of clinical criteria for functional assessment to predict primary nonfunction of high-risk livers using normothermic machine perfusion. Liver Transpl. (2018) 24(10):1453–69. 10.1002/lt.25291 30359490 PMC6659387

[46] Mergental H Perera MT Laing RW Muiesan P Isaac JR Smith A Transplantation of declined liver allografts following normothermic ex-situ evaluation. Am J Transplant. (2016) 16(11):3235–45. 10.1111/ajt.13875 27192971

[47] Watson CJE Kosmoliaptsis V Randle LV Gimson AE Brais R Klinck JR Normothermic perfusion in the assessment and preservation of declined livers before transplantation: hyperoxia and vasoplegia-important lessons from the first 12 cases. Transplantation. (2017) 101(5):1084–98. 10.1097/TP.0000000000001661 28437389 PMC5642347

[48] Nasralla D Coussios CC Mergental H Akhtar MZ Butler AJ Ceresa CDL A randomized trial of normothermic preservation in liver transplantation. Nature. (2018) 557(7703):50–6. 10.1038/s41586-018-0047-9 29670285

[49] Watson CJE Kosmoliaptsis V Pley C Randle L Fear C Crick K Observations on the ex situ perfusion of livers for transplantation. Am J Transplant. (2018) 18(8):2005–20. 10.1111/ajt.14687 29419931 PMC6099221

[50] Vogel T Brockmann JG Quaglia A Morovat A Jassem W Heaton ND The 24-hour normothermic machine perfusion of discarded human liver grafts. Liver Transpl. (2017) 23(2):207–20. 10.1002/lt.24672 27809409

[51] Eshmuminov D Becker D Bautista Borrego L Hefti M Schuler MJ Hagedorn C An integrated perfusion machine preserves injured human livers for 1 week. Nat Biotechnol. (2020) 38(2):189–98. 10.1038/s41587-019-0374-x 31932726 PMC7008032

[52] Clavien PA Dutkowski P Mueller M Eshmuminov D Bautista Borrego L Weber A Transplantation of a human liver following 3 days of ex situ normothermic preservation. Nat Biotechnol. (2022) 40(11):1610–6. 10.1038/s41587-022-01354-7 35641829

[53] Minor T Efferz P Fox M Wohlschlaeger J Luer B. Controlled oxygenated rewarming of cold stored liver grafts by thermally graduated machine perfusion prior to reperfusion. Am J Transplant. (2013) 13(6):1450–60. 10.1111/ajt.12235 23617781

[54] Hoyer DP Benko T Manka P von Horn C Treckmann JW Paul A Long-term outcomes after controlled oxygenated rewarming of human livers before transplantation. Transplant Direct. (2020) 6(4):e542. 10.1097/TXD.0000000000000987 32309628 PMC7145002

[55] Boteon YL Laing RW Schlegel A Wallace L Smith A Attard J Combined hypothermic and normothermic machine perfusion improves functional recovery of extended criteria donor livers. Liver Transpl. (2018) 24(12):1699–715. 10.1002/lt.25315 30058119 PMC6588092

[56] de Vries Y Matton APM Nijsten MWN Werner MJM van den Berg AP de Boer MT Pretransplant sequential hypo- and normothermic machine perfusion of suboptimal livers donated after circulatory death using a hemoglobin-based oxygen carrier perfusion solution. Am J Transplant. (2019) 19(4):1202–11. 10.1111/ajt.15228 30588774 PMC6590255

[57] van Leeuwen OB de Vries Y Fujiyoshi M Nijsten MWN Ubbink R Pelgrim GJ Transplantation of high-risk donor livers after ex situ resuscitation and assessment using combined hypo- and normothermic machine perfusion: a prospective clinical trial. Ann Surg. (2019) 270(5):906–14. 10.1097/SLA.0000000000003540 31633615

[58] Pegg DE. Principles of cryopreservation. Methods Mol Biol. (2007) 368:39–57. 10.1007/978-1-59745-362-2_3 18080461

[59] Jang TH Park SC Yang JH Kim JY Seok JH Park US Cryopreservation and its clinical applications. Integr Med Res. (2017) 6(1):12–8. 10.1016/j.imr.2016.12.001 28462139 PMC5395684

[60] Fahy GM Wowk B Wu J. Cryopreservation of complex systems: the missing link in the regenerative medicine supply chain. Rejuvenation Res. (2006) 9(2):279–91. 10.1089/rej.2006.9.279 16706656

[61] Fowler A Toner M. Cryo-injury and biopreservation. Ann N Y Acad Sci. (2005) 1066:119–35. 10.1196/annals.1363.010 16533923

[62] Whaley D Damyar K Witek RP Mendoza A Alexander M Lakey JR. Cryopreservation: an overview of principles and cell-specific considerations. Cell Transplant. (2021) 30:963689721999617. 10.1177/0963689721999617 33757335 PMC7995302

[63] Mazur P. The role of intracellular freezing in the death of cells cooled at supraoptimal rates. Cryobiology. (1977) 14(3):251–72. 10.1016/0011-2240(77)90175-4 330113

[64] Fuller BJ Petrenko AY Rodriguez JV Somov AY Balaban CL Guibert EE. Biopreservation of hepatocytes: current concepts on hypothermic preservation, cryopreservation, and vitrification. Cryo Lett. (2013) 34(4):432–52. 23995411

[65] Jabr F. What the supercool arctic ground squirrel teaches us about the brain’s resilience (2012). Available at: https://www.scientificamerican.com/article/arctic-ground-squirrel-brain/.

[66] Bruinsma BG Uygun K. Subzero organ preservation: the dawn of a new ice age? Curr Opin Organ Transplant. (2017) 22(3):281–6. 10.1097/MOT.0000000000000403 28266941 PMC5520671

[67] Rudolf LE Mandel S. Supercooling, intermittent perfusion, and high pressure oxygen in whole organ preservation. Transplantation. (1967) 5(4):1159–66. 10.1097/00007890-196707001-00053 4860608

[68] Usta OB Kim Y Ozer S Bruinsma BG Lee J Demir E Supercooling as a viable non-freezing cell preservation method of rat hepatocytes. PLoS One. (2013) 8(7):e69334. 10.1371/journal.pone.0069334 23874947 PMC3713052

[69] Berendsen TA Bruinsma BG Puts CF Saeidi N Usta OB Uygun BR Supercooling enables long-term transplantation survival following 4 days of liver preservation. Nat Med. (2014) 20(7):790–3. 10.1038/nm.3588 24973919 PMC4141719

[70] Sugimachi K Roach KL Rhoads DB Tompkins RG Toner M. Nonmetabolizable glucose compounds impart cryotolerance to primary rat hepatocytes. Tissue Eng. (2006) 12(3):579–88. 10.1089/ten.2006.12.579 16579691

[71] de Vries RJ Tessier SN Banik PD Nagpal S Cronin SEJ Ozer S Supercooling extends preservation time of human livers. Nat Biotechnol. (2019) 37(10):1131–6. 10.1038/s41587-019-0223-y 31501557 PMC6776681

[72] de Vries RJ Tessier SN Banik PD Nagpal S Cronin SEJ Ozer S Subzero non-frozen preservation of human livers in the supercooled state. Nat Protoc. (2020) 15(6):2024–40. 10.1038/s41596-020-0319-3 32433625 PMC8568322

[73] Huang H Yarmush ML Usta OB. Long-term deep-supercooling of large-volume water and red cell suspensions via surface sealing with immiscible liquids. Nat Commun. (2018) 9(1):3201. 10.1038/s41467-018-05636-0 30097570 PMC6086840

[74] Huang H Rey-Bedon C Yarmush ML Usta OB. Deep-supercooling for extended preservation of adipose-derived stem cells. Cryobiology. (2020) 92:67–75. 10.1016/j.cryobiol.2019.11.004 31751557 PMC7195234

[75] Sanz E Vega C Espinosa JR Caballero-Bernal R Abascal JL Valeriani C. Homogeneous ice nucleation at moderate supercooling from molecular simulation. J Am Chem Soc. (2013) 135(40):15008–17. 10.1021/ja4028814 24010583

[76] Storey KB. Life in a frozen state: adaptive strategies for natural freeze tolerance in amphibians and reptiles. Am J Physiol. (1990) 258(3 Pt 2):R559–68. 10.1152/ajpregu.1990.258.3.R559 2180324

[77] Wolanczyk JP Storey KB Baust JG. Ice nucleating activity in the blood of the freeze-tolerant frog, Rana sylvatica. Cryobiology. (1990) 27(3):328–35. 10.1016/0011-2240(90)90032-Y 2379418

[78] Tessier SN Weng L Moyo WD Au SH Wong KHK Angpraseuth C Effect of ice nucleation and cryoprotectants during high subzero-preservation in endothelialized microchannels. ACS Biomater Sci Eng. (2018) 4(8):3006–15. 10.1021/acsbiomaterials.8b00648 31544149 PMC6753837

[79] Tessier SN de Vries RJ Pendexter CA Cronin SEJ Ozer S Hafiz EOA Partial freezing of rat livers extends preservation time by 5-fold. Nat Commun. (2022) 13(1):4008. 35840553 10.1038/s41467-022-31490-2 PMC9287450

[80] Ukpai G Nastase G Serban A Rubinsky B. Pressure in isochoric systems containing aqueous solutions at subzero centigrade temperatures. PLoS One. (2017) 12(8):e0183353. 10.1371/journal.pone.0183353 28817681 PMC5560655

[81] Preciado JA Rubinsky B. Isochoric preservation: a novel characterization method. Cryobiology. (2010) 60(1):23–9. 10.1016/j.cryobiol.2009.06.010 19559692

[82] Wan L Powell-Palm MJ Lee C Gupta A Weegman BP Clemens MG Preservation of rat hearts in subfreezing temperature isochoric conditions to −8 degrees C and 78 MPa. Biochem Biophys Res Commun. (2018) 496(3):852–7. 10.1016/j.bbrc.2018.01.140 29395085

[83] Powell-Palm MJ Charwat V Charrez B Siemons B Healy KE Rubinsky B. Isochoric supercooled preservation and revival of human cardiac microtissues. Commun Biol. (2021) 4(1):1118. 10.1038/s42003-021-02650-9 34552201 PMC8458396

[84] Fahy GM MacFarlane DR Angell CA Meryman HT. Vitrification as an approach to cryopreservation. Cryobiology. (1984) 21(4):407–26. 10.1016/0011-2240(84)90079-8 6467964

[85] Boyle R Hobbes T Merret C. New experiments and observations touching cold. London: John Crook (1665).

[86] Polge C Smith AU Parkes AS. Revival of spermatozoa after vitrification and dehydration at low temperatures. Nature. (1949) 164(4172):666. 10.1038/164666a0 18143360

[87] Mazur P. Freezing of living cells: mechanisms and implications. Am J Physiol. (1984) 247(3 Pt 1):C125–42. 10.1152/ajpcell.1984.247.3.C125 6383068

[88] Pegg DE. The relevance of ice crystal formation for the cryopreservation of tissues and organs. Cryobiology. (2010) 60(3 Suppl):S36–44. 10.1016/j.cryobiol.2010.02.003 20159009

[89] Meryman HT. Mechanics of freezing in living cells and tissues. Science. (1956) 124(3221):515–21. 10.1126/science.124.3221.515 13360278

[90] Luyet BJ. The vitrification of organic colloids and of protoplasm. Biodynamica. (1937):1.

[91] Rapatz G Luyet B. Electron microscope study of erythrocytes in rapidly cooled suspensions containing various concentrations of glycerol. Biodynamica. (1968) 10(210):193–210. 5725456

[92] Rall WF Fahy GM. Ice-free cryopreservation of mouse embryos at −196 degrees C by vitrification. Nature. (1985) 313(6003):573–5. 10.1038/313573a0 3969158

[93] Sharma A Rao JS Han Z Gangwar L Namsrai B Gao Z Vitrification and nanowarming of kidneys. Adv Sci. (2021) 8(19):e2101691. 10.1002/advs.202101691 PMC8498880 34382371

[94] Gao Z Namsrai B Han Z Joshi P Rao JS Ravikumar V Vitrification and rewarming of magnetic nanoparticle-loaded rat hearts. Adv Mater Technol. (2022) 7(3). 10.1002/admt.202100873 35668819 PMC9164386

[95] Chiu-Lam A Staples E Pepine CJ Rinaldi C. Perfusion, cryopreservation, and nanowarming of whole hearts using colloidally stable magnetic cryopreservation agent solutions. Sci Adv. (2021) 7(2). 10.1126/sciadv.abe3005 33523997 PMC7793590

[96] Sharma A Lee CY Namsrai BE Han Z Tobolt D Rao JS Cryopreservation of whole rat livers by vitrification and nanowarming. Ann Biomed Eng. (2022):51. 10.1007/s10439-022-03064-2 PMC10315167 36183025

[97] Manuchehrabadi N Gao Z Zhang J Ring HL Shao Q Liu F Improved tissue cryopreservation using inductive heating of magnetic nanoparticles. Sci Transl Med. (2017) 9(379). 10.1126/scitranslmed.aah4586 28251904 PMC5470364

[98] Hossay C Donnez J Dolmans MM. Whole ovary cryopreservation and transplantation: a systematic review of challenges and research developments in animal experiments and humans. J Clin Med. (2020) 9(10). 10.3390/jcm9103196 33023111 PMC7601276

[99] Wowk B. Thermodynamic aspects of vitrification. Cryobiology. (2010) 60(1):11–22. 10.1016/j.cryobiol.2009.05.007 19538955

[100] Karow AM Jr. Cryoprotectants – a new class of drugs. J Pharm Pharmacol. (1969) 21(4):209–23. 10.1111/j.2042-7158.1969.tb08235.x 4390139

[101] Elliott GD Wang S Fuller BJ. Cryoprotectants: a review of the actions and applications of cryoprotective solutes that modulate cell recovery from ultra-low temperatures. Cryobiology. (2017) 76:74–91. 10.1016/j.cryobiol.2017.04.004 28428046

[102] Fahy GM. Cryoprotectant toxicity neutralization. Cryobiology. (2010) 60(3 Suppl):S45–53. 10.1016/j.cryobiol.2009.05.005 19501081

[103] Fahy GM Wowk B Pagotan R Chang A Phan J Thomson B Physical and biological aspects of renal vitrification. Organogenesis. (2009) 5(3):167–75. 10.4161/org.5.3.9974 20046680 PMC2781097

[104] Wusteman M Robinson M Pegg D. Vitrification of large tissues with dielectric warming: biological problems and some approaches to their solution. Cryobiology. (2004) 48(2):179–89. 10.1016/j.cryobiol.2004.01.002 15094093

[105] Burdette EC Karow AM Jeske AH. Design, development, and performance of an electromagnetic illumination system for thawing cryopreserved kidneys of rabbits and dogs. Cryobiology. (1978) 15(2):152–67. 10.1016/0011-2240(78)90020-2 668399

[106] Jackson TH Ungan A Critser JK Gao D. Novel microwave technology for cryopreservation of biomaterials by suppression of apparent ice formation. Cryobiology. (1997) 34(4):363–72. 10.1006/cryo.1997.2016 9200821

[107] Ecker HA Burdette EC Cain FL. Simultaneous microwave and HF thawing of cryogenically preserved canine kidneys. (1976).

[108] Etheridge ML Xu Y Rott L Choi J Glasmacher B Bischof JC. RF heating of magnetic nanoparticles improves the thawing of cryopreserved biomaterials. Technology. (2014) 2(3):229–42. 10.1142/S2339547814500204

[109] Gao Z Ring HL Sharma A Namsrai B Tran N Finger EB Preparation of scalable silica-coated iron oxide nanoparticles for nanowarming. Adv Sci. (2020) 7(4):1901624. 10.1002/advs.201901624 PMC7029634 32099753

[110] Boteon YL Hessheimer AJ Bruggenwirth IMA Boteon A Padilla M de Meijer VE The economic impact of machine perfusion technology in liver transplantation. Artif Organs. (2022) 46(2):191–200. 10.1111/aor.14131 34878658

[111] Watson CJE Hunt F Messer S Currie I Large S Sutherland A In situ normothermic perfusion of livers in controlled circulatory death donation may prevent ischemic cholangiopathy and improve graft survival. Am J Transplant. (2019) 19(6):1745–58. 10.1111/ajt.15241 30589499

[112] Barrou B Billault C Nicolas-Robin A. The use of extracorporeal membranous oxygenation in donors after cardiac death. Curr Opin Organ Transplant. (2013) 18(2):148–53. 10.1097/MOT.0b013e32835e29f5 23385885

[113] Kidney transplantation: past, present, and future. Available at: https://web.stanford.edu/dept/HPST/transplant/html/history.html.

[114] The official Dr. Thomas E. Starzl web site. Available at: https://www.starzl.pitt.edu/about/starzl.html.

[115] Belzer F. Organ preservation: a personal perspective. Available at: https://web.stanford.edu/dept/HPST/transplant/html/belzer.html.

[116] Clarke A. Is there a universal temperature dependence of metabolism? Funct Ecol. (2004) 18(2):252–6. 10.1111/j.0269-8463.2004.00842.x

